# Successful continuous-flow left ventricular assist device implantation with adjuvant tricuspid valve repair for advanced heart failure

**DOI:** 10.5830/CVJA-2016-034

**Published:** 2016

**Authors:** Chih-Hsien Lee, Jeng Wei

**Affiliations:** Department of Cardiac surgery, tungs’ taichung metroharbor hospital; department of Biological science and technology, National Chiao-tung university; department of surgery, National defense medical Centre, taiwan; Heart Centre, Cheng hsin General hospital, Taiwan

**Keywords:** cf-LVAD, end-stage heart failure, HeartMate II

## Abstract

The prevalence of end-stage heart failure (HF) is on the increase, however, the availability of donor hearts remains limited. Left ventricular assist devices (LVADs) are increasingly being used for treating patients with end-stage HF. LVADs are not only used as a bridge to transplantation but also as a destination therapy. HeartMate II, a new-generation, continuous-flow LVAD (cf-LVAD), is currently an established treatment option for patients with HF. Technological progress and increasing implantation of cf-LVADs have significantly improved survival in patients with end-stage HF. Here we report a case of a patient with end-stage HF who was successfully supported using cf-LVAD implantation with adjuvant tricuspid valve repair in a general district hospital.

## Abstract

HeartMate II is a new-generation, continuous-flow left ventricular assist device (cf-LVAD, Thoratec, Pleasanton, CA, USA) used as a bridge to transplantation (BTT) and as a destination therapy (DT) in patients with end-stage heart failure (HF).^1^ Applying continuous-flow technology to mechanical circulatory support systems has revolutionised the treatment of end-stage HF.

The use of implantable mechanical circulatory support devices, such as cf-LVAD, has increased in recent years.^2^ Safer long-term cf-LVAD support has been achieved because of improved outcomes.^1^ Patients supported for BTT or DT using cf-LVADs have an overall reduction in life-threatening complications and have prolonged survival time, with an active lifestyle and an acceptable quality of life. Cardiac transplant recipients can safely wait for extended periods while their status at transplant is optimised, aiding post-transplant survival. In this case report, we describe a patient with end-stage HF who was successfully supported using cf-LVAD implantation with adjuvant tricuspid valve repair.

## Case report

A 39-year-old man was admitted to our hospital because of deteriorating heart function despite maximal medical treatment. He had a body surface area of 1.88 m^2^, had suffered from advanced HF following dilated cardiomyopathy, and had been waiting for a heart transplant for four years. In addition, he had diabetes mellitus requiring insulin control. On examination, he exhibited bilateral grade IV pitting oedema of the lower limbs with bilateral pleural effusion and ascites.

Laboratory tests showed a total bilirubin level of 6.2 mg/dl, aspartate aminotransferase level of 109 U/l, and blood creatinine level of 1.8 mg/dl. Echocardiography revealed a left ventricular (LV) diastolic diameter of 67 mm, LV systolic diameter of 58 mm, and ejection fraction of 19% with severe tricuspid regurgitation (TR).

A pulmonary artery catheter was inserted to measure pulmonary artery systolic pressure, pulmonary capillary wedge pressure, central venous pressure and cardiac index, which were found to be 34, 50 and 36 mmHg, and 0.5 l/min/m^2^, respectively. This facilitated optimising the use of dobutamine and dopamine to decrease the right ventricular (RV) afterload while maintaining ventricular contractility. An intra-aortic balloon pump was inserted at the same time because the urine output had decreased. Furosemide was continuously administered to obtain an adequate urine output and to decrease central venous pressure to < 24 mmHg. Right ventricular failure (RVF) is common after cf-LVAD implantation and is a leading cause of morbidity and death after cf-LVAD implantation.

Five days after admission, tricuspid valve repair was performed using the de Vega annuloplasty procedure under anaesthesia. HeartMate II was implanted under cardiopulmonary bypass. The alignment of the mitral valve and inflow cannula was checked using transoesophageal echocardiography.

Initial pump flow was at 6 000 rpm, and we gently needlepunctured the cf-LVAD outflow tract to assist in de-airing the intracardiac air bubbles. The needle and clamp were removed, cardiopulmonary bypass was terminated, and the pump speed was increased from 6 000 to 9 200 rpm; the pump flow was 4.5 l/m, pulse index was 5.7, pump power was 5.5 W, and mean blood pressure was 60 mmHg. Protamine was administered slowly. The intra-aortic balloon pump was removed, and an open repair of the left femoral artery was performed. Dobutamine and dopamine maintained the RV contractility and decreased the RV afterload.

The patient’s condition stabilised, and he was transferred to the intensive care unit. The endotracheal tube was removed the next day. More than 12 hours following cf-LVAD implantation, when the chest tube drainage decreased to ≤ 50 ml/h and the coagulation profile returned to normal levels, an intravenous heparin infusion was started to maintain activated partial thromboplastin time between 50 and 70 s. Aspirin (100 mg) was administered once daily after extubation, and warfarin was administered to maintain the international normalised ratio (INR) between 2.0 and 3.0. Heparin infusion was continued until the INR target range was attained. He was discharged one month after the operation and was categorised as New York Heart Association functional class II.

## Discussion

The prevalence of end-stage HF is on the increase, however, the availability of donor hearts remains limited. Therefore, the number of patients requiring long-term support with cf-LVAD implantation has increased. HeartMate II is a new-generation cf-LVAD used as BTT and DT in patients with end-stage HF.^1^,^3^

The waiting time for cardiac transplant recipients has increased, and so has BTT by using the support device in clinical settings. We occasionally encounter patients who require unexpected long-term device support. In addition to being a life-saving treatment, cf-LVADs currently also provide long-term survival with favourable quality of life for patients with severe HF. Consequently, long-term cf-LVAD implantation has become a valuable alternative to cardiac transplantation for treating end-stage HF.

Studies have reported that post-transplant survival at one, two, five and 10 years is approximately 90, 80, 70 and 50%, respectively.^1^ The survival of patients receiving DT with cf-LVADs within this cohort at one, three and five years was 80–83, 75 and 61%, respectively.^1^,^4^

Prolonged post-transplant survival in patients receiving BTT can reduce the need for cardiac transplantation as a first-line replacement therapy. However, it is too premature to draw conclusions about survival comparisons because of the lack of head-to-head comparative data. Furthermore, considering the frequent readmissions in this population, patient survival, quality of life, and healthcare costs should be considered before drawing conclusions.^1^ Current cf-LVADs provide satisfactory long-term survival, but rehospitalisation for specific reasons is common in this population.^1^

Despite this progress, cf-LVAD implantation is still associated with a risk of complications, which challenges patient selection and adversely impacts on outcomes. The incidence of RVF in this population ranges from 10 to 50% and is a risk factor for peri-operative and postoperative mortality and morbidity in patients undergoing cf-LVAD implantation.^2^,^4^,^5^

Moreover, some reports have suggested that cf-LVAD implantation in patients with pre-operative hepatic failure entails considerable mortality.2 cf-LVAD recipients who develop postoperative RVF have poor outcomes, including increased incidences of multi-organ failure, postoperative haemorrhage, pulmonary complications, thromboembolic events, and migration of intracardiac air to the coronary arteries, causing transient myocardial ischaemia.^5^ In this setting, the function of the right heart becomes critical to patient survival, and RVF remains a considerable postoperative complication that affects mortality.

In a previous study, 33.4% of patients experienced RVF postoperatively and 10–15% required RV support.^2^,^5^ RVF after cf-LVAD implantation is occasionally unavoidable in a deteriorated heart. Therefore RVF is a contraindication for receiving cf-LVAD implantation because it may require the use of a biventricular assist device. The setting of RVF is associated with a poor prognosis and influences early outcomes.^5^

The prediction and treatment of RVF are crucial to improve survival after cf-LVAD implantation. The ratio of central venous pressure to pulmonary capillary wedge pressure and secondary pulmonary hypertension is a critical predictor of RVF after cf-LVAD implantation; RVF significantly reduces survival after cf-LVAD implantation.^5^ Careful evaluation of central venous pressure, pulmonary capillary wedge pressure, and laboratory data may help to predict postoperative RVF.

Furthermore, secondary TR is common among patients with RVF who undergo cf-LVAD implantation. Although the repair of TR in combination with cf-LVAD implantation is not an established approach, recent reports have suggested that concomitant tricuspid valve repair may reduce postoperative RVF.^5^ In our case, tricuspid valve repair was performed by the de Vega annuloplasty procedure to decrease the risk of RVF. To determine whether tricuspid valve repair is useful to rescue patients from possible RVF, a randomised study is required.

HeartMate II may transiently worsen the right ventricular function in the initial post-implant period because of a higher cardiac output resulting in increased venous return and right ventricular preload.^2^ In addition, this initial temporary cholestasis resulting from RV dysfunction has been documented previously.^2^ Although laboratory parameters tend to normalise with successful cf-LVAD implantation, the effect appears to dissipate over time. A gradual improvement in RVF caused by improved LV unloading is observed in the majority of patients.

Survival rates have increased because major adverse events, such as stroke, bleeding and infection, have decreased. The occurrence of thrombosis ranges from annualised rates of 2–4% and that of haemolysis ranges from 2–3%.^3^ The major highrisk factors, such as female gender, young age, implantation technique, and inflow cannula malposition, are related to the development of pump thrombosis.^3^ Other risk factors, such as sub-therapeutic INR, non-compliance, hypercoagulable disorder, and infection require pre-operative optimisation, intraoperative techniques, and postoperative management strategies.^3^ The pre-operative period of haemodynamic and fluid balance is optimised when possible.

Some reports have evaluated heparin antibodies, aspirin resistance and baseline platelet function where possible.^3^ An adequate pump pocket depth is critical to allow favourable positioning of the cf-LVAD and inflow cannula angle, which should lie parallel to the septum and oriented to the central LV and mitral valve. Echocardiography is essential to enable surgeons and anesthaesiologists to make prompt decisions during cf-LVAD implantation and is necessary for detecting cardiac structural and functional abnormalities, such as ventricular dysfunction, valvular pathology, mural thrombosis, and atrial septal defect or patent foramen ovale. The pocket is made deep and lateral to allow the pump to be fixed below the diaphragm. The outflow cannula is placed to the right of the sternal midline with enough graft length to avoid any compression of the RV.

The management of bleeding is indivisibly linked to the risk of thromboembolic events, and anticoagulation and antiplatelet therapies seem to be the only methods for carefully managing complication and individual risks. Further understanding of the mechanisms underlying bleeding and novel strategies, such as new anticoagulant drugs, are expected to play crucial roles in the long-term management of cf-LVAD therapy.^1^

Cardiac arrhythmia, such as ventricular arrhythmia, is also a common issue in the early and late periods after cf-LVAD implantation. Although such arrhythmias may not be lethal in the presence of cf-LVAD, it could put patients at a risk of RVF.^1^ Anti-arrhythmic medication, catheter ablation, intra-operative cryoablation, and implantable cardioverter–defibrillator may be employed to minimise the risk of recurrent arrhythmias.^1^

End-organ function was restored one month after the initiation of support.^1^ These improvements persisted throughout the support period; for example, the LV diastolic dimension significantly decreased and the TR ratio reduced from 45 to 22% at one month, except for the creatinine level.^1^

## Conclusion

The implantation of cf-LVAD, either as BTT or DT, remains a critical treatment option for selected patients with end-stage HF.
